# Strategic tuning of precursor's concentration for the synthesis of Sb_2_S_3_ thin films with enlarged nanocrystals and *hk*1-oriented growth, leading to superior optical properties

**DOI:** 10.1039/d5na00901d

**Published:** 2025-11-12

**Authors:** Md Abrar Faisal Hossain, Kyota Shirai, Masayuki Shimojo

**Affiliations:** a Department of Mechanical Engineering, The Hong Kong Polytechnic University 11 Yuk Choi Road Hung Hom Hong Kong China abrar.hossain@connect.polyu.hk +852 6683 7589; b Department of Materials Science and Engineering, Shibaura Institute of Technology 3-7-5 Toyusu Koto Tokyo 135-8548 Japan

## Abstract

Antimony trisulfide (Sb_2_S_3_) has acquired significant attention due to its non-toxic nature, durability, abundance, and superior opto-electronic properties, making it a promising candidate for various applications in optoelectronics and photovoltaics. It is important to focus on achieving the desired crystal morphology and preferred growth (*hk*1-oriented), as these can potentially result in superior optical properties. Moreover, surface morphology and stoichiometric ratio of the fabricated films also play a significant role. Even after conducting intensive research to address these factors, there remains immense research scope to enhance these properties. In this study, we present a strategically optimized precursor concentration for solution-based synthesis of Sb_2_S_3_ thin films with large, compact, homogeneous nanocrystals, *hk*1-oriented preferential growth and superior optical properties, through a simple, cost-effective spin coating method. By varying the concentration of CS_2_ (source of sulphur), an enhanced precursor solution was synthesized, which yielded the desirable crystal dimensions, *hk*1-oriented growth and optical properties. In this research work, we demonstrate the results of varying the concentration of CS_2_ (from 1.5 ml to 3 ml with steps of 0.5 ml) and the effect it has on the overall structural and optical properties. The synthesized materials were characterized comprehensively using High-Resolution Transmission Electron Microscopy (HR-TEM), Bright-Field Transmission Electron Microscopy (BF-TEM), Selected Area Electron Diffraction (SAED), Scanning Electron Microscopy (SEM), Energy-Dispersive X-ray Spectroscopy (EDS), and Variable Angle Spectroscopic Ellipsometry (VASE). Our experimental findings of BF-TEM, HR-TEM, SAED, and VASE conclude that the precursor solution consisting of 2.5 ml of CS_2_ was able to generate enhanced nanocrystals, *hk*1-oriented growth and superior optical properties. The other measurements taken using the characterizing techniques agreed well with the findings of BF-TEM, HR-TEM, SAED, and VASE.

## Abstract 2

Sb_2_S_3_ thin films fabricated with *hk*1-oriented crystal growth and enlarged nanocrystals lead to superior optical properties, with great promise for potential integration into photovoltaic devices and future optoelectronic applications.

## Graphical abstract

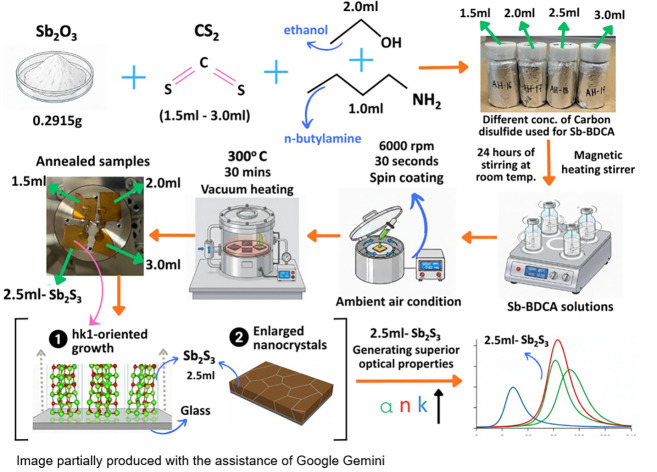

## 1. Introduction

Metal chalcogenide (MC) semiconductors of type M_2_X_3_ (where M = As, Sb, Bi; X = S, Se, Te) have particularly gained substantial research interest recently due to their applications in photovoltaics,^1–4^ thermoelectrics,^5–7^ optoelectronics,^8–11^ memory devices,^12,13^ photocatalysis^14^ and neuromorphic implementation.^15^ Among such M_2_X_3_ semiconductor materials, antimony trisulfide (Sb_2_S_3_) with a quasi-one-dimensional ribbon like structure has shown significant promise due to its excellent photosensitivity and thermoelectric power,^16^ specified quantum size effects,^17^ high absorption coefficient (∼10^5^ cm^−1^ over the UV-visible range)^18^ and refractive index,^19^ optimum optical band gap (around 1.7 eV),^20^ along with low toxicity, ample abundancy and great durability. Owing to these properties, antimony trisulfide has found its way into inorganic heterojunction, hybrid and dye-sensitized solar cells.^21–23^ Sb_2_S_3_ has also proven to be a potential material for applications in thermoelectric cooling, optoelectronics, microwave devices and television cameras.^24^

To date, there have been many synthesis and deposition routes suggested by researchers for fabricating Sb_2_S_3_ thin films, which can roughly be categorized into vacuum-assisted and solution-processed methods. Vacuum-assisted methods may include Rapid Thermal Evaporation (RTE), Vapor Transport Deposition (VTD), Close Space Sublimation (CSS), and magnetron sputtering.^25^ Solution-processed methods include Hydrothermal Deposition (HD), spin-coating, and Chemical Bath Deposition (CBD).^18^ Solution-processed synthesis methods have achieved considerable interest due to their low cost, ease of fabrication, rapid production, scope of large-area manufacturing, and most importantly their ability to generate large grain sized films, thereby reducing the grain boundary density and improving the device performance.^26^ Among the solution-processed methods, the most prominent deposition technique is regarded to be the spin-coating method, as other methods like CBD result in a greater generation of chemical waste, are time-intensive, and produce high impurity levels, while HD necessitates a more costly experimental setup.^27^ Solution-processed methods generally include the development of a precursor solution, which eventually leads to the formation of the thin film. Depending on the precursor formulation, a suitable method of fabricating (CBD, HD, or spin-coating) the thin film is chosen. The precursor solution synthesis route proposed by Wang *et al.*,^28^ an easy one-step precursor formulating method which involves dissolving Sb_2_O_3_ in ethanol-diluted butyldithiocarbamic acid (BDCA), is also applied in this work with deliberate modifications.

Surface defects, unfavourable *hk*0 orientations, and high grain boundary density are the major setbacks commonly faced in fabricating thin films of metal chalcogenides. Thus far, there have been many efforts made to tackle such challenges *via* engineering precursor molar ratios,^27,29,30^ introducing additional sulphur sources during performing Chemical Bath Deposition (CBD) and Vertical Transport Deposition (VTD),^31,32^*in situ* addition of Cd^2+^ ions or tartaric acid during Hydrothermal Deposition (HD),^33,34^ and performing post-treatment of the film by spin-coating an SbCl_3_ layer on Sb_2_S_3_.^35^ Such efforts were made to achieve an ideal stoichiometric ratio (Sb : S, 2 : 3), uniform morphology mitigating defects and pinholes, favourable *hk*1 orientation crystal planes, and a large grain size with reduced grain boundary density. However, most of the vacuum-assisted (VTD and CSS) and some solution-processed (CBD and HD) methods do not allow complete control over the film growth and stoichiometric ratio, making it difficult to address the major film fabrication challenges. Thus, there remains opportunity for improvement in potential film fabrication techniques to achieve stable crystal growth, uniform surface morphology, favourable *hk*1-oriented crystal planes, and reduced grain boundary density.

In this research work, we conducted a comprehensive study on the structural, morphological, compositional, and optical aspects of Sb_2_S_3_ thin films synthesized *via* a strategically tuned concentration precursor solution. We adopted the precursor solution synthesis route proposed by Wang *et al.*^28^ with some deliberate tactical modifications to the concentration of one of the reactants to obtain enhanced film characteristics. As mentioned by Chen *et al.*,^18^ solution-processed methods generally yield a reduced grain boundary density compared to vacuum-assisted methods like VTD, CSS, and sputtering. By implementing our strategic precursor solution route, we can deposit our thin film *via* a simple, cost-effective spin coating technique. Our recipe for the precursor solution included antimony trioxide (Sb_2_O_3_) as the antimony source and carbon disulfide (CS_2_) as the carbon source, along with ethanol and butylamine. We carefully increased the CS_2_ concentration from 1.5 ml to 3 ml with steps of 0.5 ml, keeping other reactants constant. The thin films fabricated were extensively characterized to study various properties of the deposited thin films.

## 2. Experimental methods

### 2.1. Chemicals

Antimony trioxide (FUJIFILM, 95%), carbon disulfide (FUJIFILM, 99%), and *n*-butylamine (FUJIFILM) were purchased from FUJIFILM Wako Pure Chemical Corporation and used without further purification.

### 2.2. Preparation of antimony trisulfide (Sb_2_S_3_) precursor solution

The Sb_2_S_3_ precursor solution, which is essentially a metal–acid (metal–BDCA) complex solution, was prepared following the recipe proposed by Wang *et al.*,^28^ with some strategic modifications to it. 1 mmol (0.2915 g of Sb_2_O_3_) of antimony trioxide (Sb_2_O_3_) was added to each of the four empty 20 ml glass vials, followed by 2 ml ethanol. Afterwards 1.5–3.0 ml CS_2_ was added in steps of 0.5 ml to produce four distinct precursor solutions. At last, 1 ml of butylamine was added to each of the four solutions. The solution mixtures were stirred overnight for 24 hours to produce a clear solution. For simplicity, from here on, the films produced by these solutions will be referred to as 1.5-Sb_2_S_3_, 2.0-Sb_2_S_3_, 2.5-Sb_2_S_3_, and 3.0-Sb_2_S_3_ as appropriate.

### 2.3. Film fabrication and characterization

The obtained precursor solutions were spin-coated on borosilicate glass substrates (purchased from Matsunami) at 6000 rpm for 30 seconds. Before using the glass substrates, they were thoroughly cleaned using an ultrasonic cleaner, with acetone and ethanol, followed by DI water. Upon spin-coating, all the samples were annealed in a vacuum at 300 °C for 30 minutes to obtain a carbon-free crystallized Sb_2_S_3_ thin film.

High-Resolution Transmission Electron Microscopy (HR-TEM), Bright-Field Transmission Electron Microscopy (TEM), and Selected Area Electron Diffraction (SAED) were performed to obtain High-Resolution (HR) images, Bright-Field (BF) images, and selected area electron diffraction patterns, respectively, using a TEM JEM 2100 (JEOL). Scanning Electron Microscopy (SEM) was performed using a Hitachi S-4500 to observe the surface morphology. Energy Dispersive Spectroscopy (EDS) coupled with SEM was performed using a W-SEM JSM-IT510LA (JEOL) to obtain the chemical composition. A variable angle spectroscopic ellipsometer (RC2-DI-SUY) was used to obtain the optical properties of the thin film.

## 3. Results and discussion

The annealed films (1.5-Sb_2_S_3_, 2.0-Sb_2_S_3_, 2.5-Sb_2_S_3_, and 3.0-Sb_2_S_3_) were characterized thoroughly to study and analyse their properties. The obtained results from the characterization process are demonstrated in this section. All the data achieved conclude that 2.5-Sb_2_S_3_ obtained superior structural and optical properties.

### 3.1. Bright-field TEM and high-resolution TEM

**Fig. 1:**
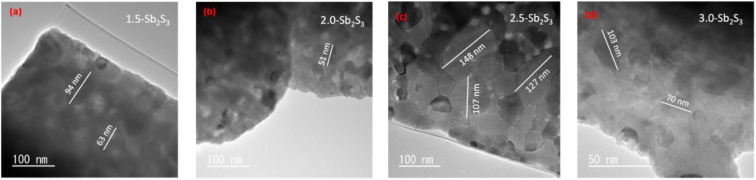
Bright-field TEM images of the Sb_2_S_3_ thin films annealed at 300 °C: (a) 1.5-Sb_2_S_3_, (b) 2.0-Sb_2_S_3_, (c) 2.5-Sb_2_S_3_, and (d) 3.0-Sb_2_S_3_.

**Fig. 2:**
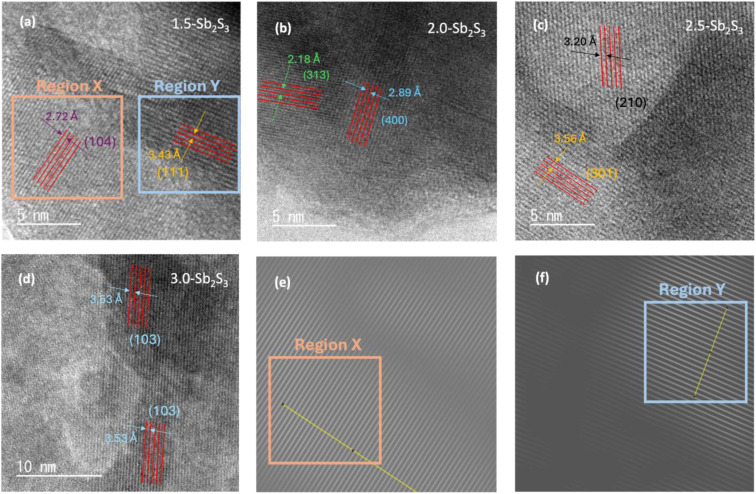
HR-TEM images of the Sb_2_S_3_ thin films annealed at 300 °C: (a) 1.5-Sb_2_S_3_, (b) 2.0-Sb_2_S_3_, (c) 2.5-Sb_2_S_3_, and (d) 3.0-Sb_2_S_3_. FFT-diffractogram images of 1.5-Sb_2_S_3_ in (e) region X and (f) region Y.

Bright-field TEM and high-resolution TEM images of the 4 samples (1.5-Sb_2_S_3_, 2.0-Sb_2_S_3_, 2.5-Sb_2_S_3_, and 3.0-Sb_2_S_3_) acquired with a TEM are shown in Fig. 1 and 2, respectively. Solution-processed and spin-coated samples are often difficult to analyse with a TEM due to the complexity associated with transferring the sample to the TEM mesh/grid. In this research, we adopted adhering the TEM mesh on the glass substrate using carbon tape and then performing the spin coating on the mesh/glass substrate. After completion of spin coating, the carbon tape was peeled off, and the mesh was heated in a vacuum for 30 minutes at 300 °C. This TEM sample preparation method ensured that the film was free of any physical contact and provided reliable, accurate results. The bright-field TEM images obtained with an accelerating voltage of 200 kV are shown in Fig. 1, which reveal the nanocrystalline structure of the fabricated thin films. 2.5-Sb_2_S_3_ (Fig. 1(c)) shows more uniform crystal thickness compared to 1.5-Sb_2_S_3_ (Fig. 1(a)) and 2.0-Sb_2_S_3_ (Fig. 1(b)), and 2.5-Sb_2_S_3_ has much larger crystals (∼120 nm) (Fig. 1(c)) than 1.5-Sb_2_S_3_ (∼70 nm) (Fig. 1(a)), 2.0-Sb_2_S_3_ (∼50 nm) (Fig. 1(b)), and 3.0-Sb_2_S_3_ (∼90 nm) (Fig. 1(d)). Incorporating 1.5 ml and 2.0 ml of CS_2_ proved to be insufficient to saturate all nucleation sites; as a result, some region remained underdeveloped, leading to local variations in crystal thickness. However, some tiny white particles can be observed along the boundaries, which are likely residual nuclei of Sb_2_S_3_.^28^Fig. 1(a) and (b) can be observed to have similar morphological properties with non-uniform thickness and smaller crystals. Therefore, increasing the CS_2_ concentration to 2.5 ml yielded larger nanocrystals (nearly twice the size of 1.5-Sb_2_S_3_ and 2.0-Sb_2_S_3_) and more uniform crystal thickness.

High-resolution TEM images were taken using a TEM to observe the overall morphology of the crystal lattice as shown in Fig. 2. HR-TEM images of the four samples show visible lattice fringes confirming the crystallinity of the samples. Some differences in uniformity can be observed, which shows the relative overlapping of different lattice fringes. No evident crystal defects such as dislocation of the lattice fringes can be observed from the high-resolution TEM images at a resolution of 5–10 nm. Hence, all the fabricated films possessed well-resolved lattice fringes without any obvious evidence of structural defects. Furthermore, to analyse the crystal plane, Fast Fourier Transform (FFT) was performed on the lattice fringes with the help of ImageJ software. This helped to measure the interplanar spacing (*d*-spacing) of the lattice accurately. FFT-diffractogram images of lattice fringes of 1.5-Sb_2_S_3_ are shown in Fig. 2(e) (representing region “X”) and Fig. 2(f) (representing region “Y”). Measuring the *d*-spacing of these lattice fringes revealed *d* = 2.72 Å (104) for region X and *d* = 3.43 Å (111) for region *Y*. Similarly, FFT diffraction was also performed on other samples. 2.0-Sb_2_S_3_ showed *d* = 2.18 Å (313) and *d* = 2.89 Å (400), Fig. 2(b). 2.5-Sb_2_S_3_ fringes (Fig. 2(c)) demonstrated interplanar *d*-spacings of *d* = 3.20 Å (210) and 3.56 Å (301). Lastly, 3.0-Sb_2_S_3_ showed *d* = 3.53 Å (103). Therefore, high-resolution TEM images confirmed the crystallinity across all the samples with no evident defects (dislocations), whereas bright-field TEM images revealed the large crystal sizes of 2.5-Sb_2_S_3._

### 3.2. Scanning electron microscopy (SEM)

**Fig. 3:**
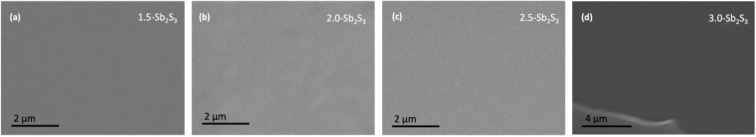
Top-view SEM images of the Sb_2_S_3_ thin films annealed at 300 °C: (a) 1.5-Sb_2_S_3_, (b) 2.0-Sb_2_S_3_, (c) 2.5-Sb_2_S_3_ and (d) 3.0-Sb_2_S_3_.

Scanning electron microscopy was performed to understand the surface morphology of the annealed thin films. Fig. 3(a)–(d) show the obtained top-view SEM images of the samples. All the SEM images display a compact, homogeneous surface morphology at the micron scale (around 2 µm). No visible grain boundaries could be seen at such a scale, which disregards the presence of any dense grain boundary region. This shows that the surface is free of any suppressing defect states and ensures efficient charge transport, which is significant for photovoltaic applications. The absence of large and visible cracks and pinholes at such a scale confirms the uniform, compact, spin-coated and annealed films. These findings support the superior surface morphology of the fabricated films, only at the micron scale. It is worth noting that the parameters related to SEM measurements (accelerating voltage, magnification) were adjusted to certain values but did not produce any visible boundaries or defects. Hence, the top-view SEM images of the fabricated thin films show impressively compact and homogeneous surface morphology without the presence of visible dense grain boundaries.

### 3.3. Selected area electron diffraction (SAED) pattern

**Fig. 4:**
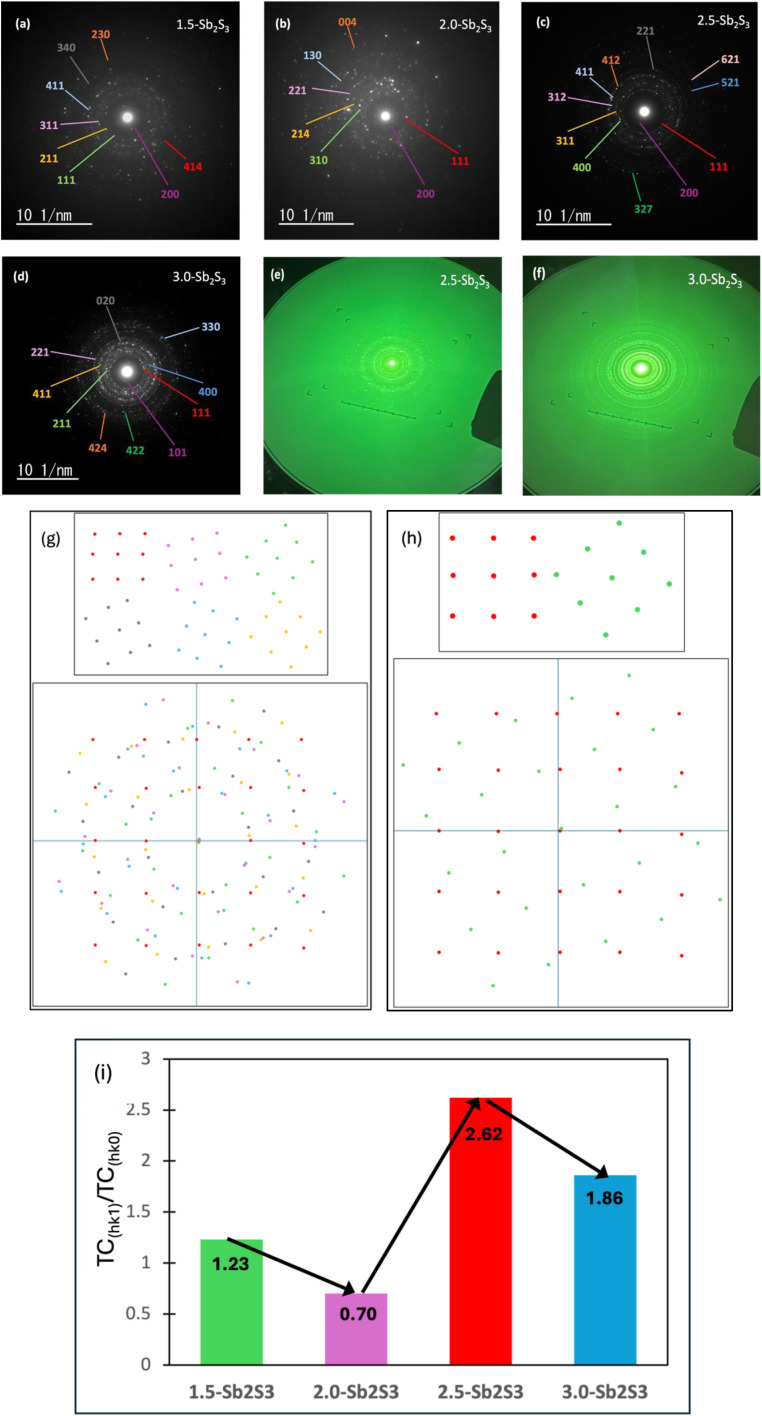
SAED pattern images of the Sb_2_S_3_ thin films: (a) 1.5-Sb_2_S_3_, (b) 2.0-Sb_2_S_3_, (c) 2.5-Sb_2_S_3_ and (d) 3.0-Sb_2_S_3_. Electron diffraction images of (e) 2.5-Sb_2_S_3_ and (f) 3.0-Sb_2_S_3_, captured with an external camera. Illustration of diffraction patterns of dense (g) and sparse (h) regions. (i) Texture coefficients (TC_(*hk*1)_/TC_(*hk*0)_).

The SAED pattern was performed using a TEM to identify the orientation of the crystal planes (Miller indices) across a selected area of nanocrystals and to analyse the corresponding diffraction spots. SAED patterns can reveal crystal planes (rings corresponding to XRD peaks), which cannot be observed in XRD for nanocrystalline films. Fig. 4(a)–(d) show the SAED patterns obtained for 1.5-Sb_2_S_3_, 2.0-Sb_2_S_3_, 2.5-Sb_2_S_3_, and 3.0-Sb_2_S_3_, respectively. The SAED patterns of all the samples show bright diffraction spots, and the absence of any diffused rings proves that there are no amorphous regions. Hence, all the samples obtained show a pure crystalline phase with no amorphous regions. It is important to note that the observation area used in generating the SAED patterns were constant across all the samples. Hence, when a dense diffraction pattern is obtained, it refers to the existence of many small crystals with different orientations, in the area selected for diffraction. This concept is illustrated in Fig. 4(g), where six differently oriented crystals produce a dense diffraction pattern. In contrast, when a sparse diffraction pattern is obtained, it refers to the existence of lower number of crystals and of bigger size, as the selected area aperture was the same. This is illustrated in Fig. 4(h). Following these concepts, it can be concluded that 2.0-Sb_2_S_3_ (Fig. 4(b)) and 3.0-Sb_2_S_3_ (Fig. 4(d)) possessed smaller crystals as the SAED patterns showed some areas of dense diffraction spots. Interestingly, the SAED pattern of 2.5-Sb_2_S_3_ (Fig. 4(c)) also shows some region of dense diffraction pattern, revealing the presence of smaller crystals. This could be explained by the BF-TEM findings of 2.5-Sb_2_S_3_, which show bigger (∼130 nm) and also smaller (∼60 nm) crystals.

The Miller indices (*hkl*) representing the orientation of the crystal planes were calculated using eqn (1) for a polycrystalline orthorhombic structure, where *d* is the interplanar spacing and *a*, *b*, and *c* are the lattice constants with values *a* = 11.31 Å, *b* = 3.83 Å, and *c* = 11.23 Å.1
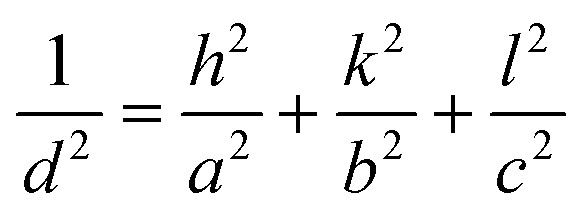


The value of interplanar spacing (*d*) was calculated by measuring the diameter (*D*) of the ring, followed by the radius (*r* = *D*/2), and then taking the reciprocal of the radius (1/*r*). All the units were converted to Ångström (Å) before starting the calculation. Different values of *h*, *k*, and *l* (in ascending order) were fit to eqn (1), and the values that generated the least error were selected. When looking at the Miller indices, it could be seen that the samples have preferential growth in the *hk*1 direction proven by the texture coefficients (TC) (*hk*1/*hk*0) shown in Fig. 4(g), which is beneficial for optoelectronic devices to achieve high efficiency.^18^ It is worth noting that solution-processed spin-coated samples usually tend to favour *hk*0-oriented growth of the Sb_4_S_6_ nanoribbons; however, in our research work, we have successfully demonstrated *hk*1 dominated growth *via* the solution-processed spin coating method.^18^Fig. 4(g) shows that 2.5-Sb_2_S_3_ obtained the highest texture coefficient (2.62 (*hk*1/*hk*0)) among all the samples, clearly indicating its superior *hk*1 growth characteristic. The pronounced *hk*1-oriented growth observed in the 2.5-Sb_2_S_3_ sample can be linked to an optimal balance between sulphur availability and nucleation kinetics. Tuning the CS_2_ concentration in the precursor solution to 2.5 ml resulted in a sufficient supply of sulphur to saturate nucleation sites and promote crystal growth in the desired *hk*1 orientation. Increasing the concentration of CS_2_ further to 3.0 ml drives the system to enter a state of supersaturation, which leads to rapid, uncontrolled nucleation. This results in the formation of smaller nanocrystals (proven by the dense diffraction spots and bright-field TEM images) instead of continued growth of existing crystals, producing non-uniform orientations and a reduction of *hk*1-oriented growth. However, both 2.5-Sb_2_S_3_ (c) and 3.0-Sb_2_S_3_ (d) show favourable *hk*1 oriented film growth (2.62 and 1.86, respectively) compared to 1.5-Sb_2_S_3_ (a) and 2.0-Sb_2_S_3_ (b) (1.23 and 0.70, respectively). The lower TC values obtained for 1.5-Sb_2_S_3_ and 2.0-Sb_2_S_3_ could be attributed to the unavailability of sulphur, for promoting *hk*1 growth. The Miller indices (101), (111), (211), (221), (311), and (411) match well with other research papers.^20,31,32,36–38^ Hence, the TC measurements show that the 2.5-Sb_2_S_3_ (Sb_4_S_6_)_*n*_ ribbons achieved the overall highest texture coefficients (TC_(*hk*1)_/TC_(*hk*0)_). Images in Fig. 4(e) and (f) were captured with an external camera to display the SAED pattern rings visible to the naked eye to confirm the crystallinity as these specific images were unable to be captured with the TEM camera due to its chances of getting burnt. Therefore, the TCs measured from the SAED pattern of the fabricated films revealed that tuning of the precursor's concentration can generate Sb_2_S_3_ films with *hk*1-oriented crystal growth.

### 3.4. Energy dispersive X-ray spectroscopy (EDS)

**Fig. 5:**
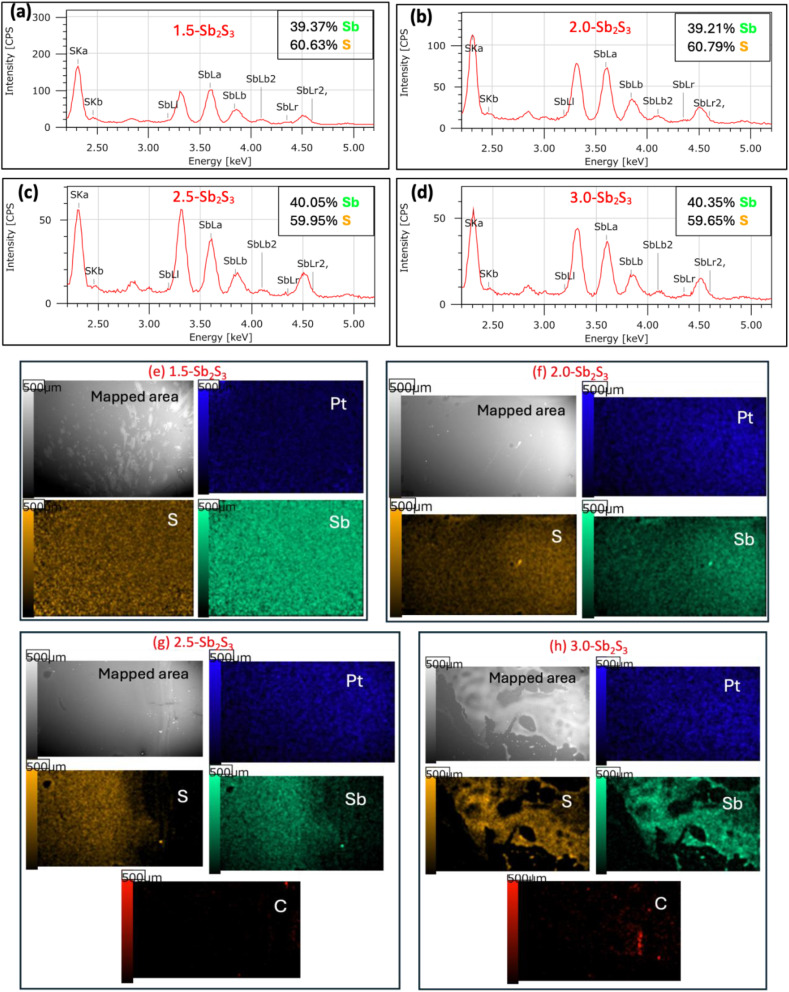
Energy dispersive X-ray spectroscopy (EDS) compositional analysis of (a) 1.5-Sb_2_S_3_, (b) 2.0-Sb_2_S_3_, (c) 2.5-Sb_2_S_3_ and (d) 3.0-Sb_2_S_3_. Elemental distribution analysis of (e) 1.5-Sb_2_S_3_, (f) 2.0-Sb_2_S_3_, (g) 2.5-Sb_2_S_3_ and (h) 3.0-Sb_2_S_3_.

An energy dispersive X-ray spectrometer equipped with a scanning electron microscope was employed to determine the elemental composition and distribution of the synthesized Sb_2_S_3_ samples and to verify their chemical stoichiometry. An accelerating voltage of 20.00 kV, working distance of 10.5 mm, (×30) magnification and high vacuum mode were utilized to perform EDS. The atomic percentage was evaluated by composition analysis, and the elemental distribution was found by performing area mapping. Fig. 5(a)–(d) show that all 4 samples (1.5-Sb_2_S_3_, 2.0-Sb_2_S_3_, 2.5-Sb_2_S_3_, and 3.0-Sb_2_S_3_, respectively) reached the ideal stoichiometric ratio of 2 : 3 (Sb : S).^19,39^ The change in concentration of the CS_2_ in the precursor solution did not seem to affect the end stoichiometric ratio much, whereas in other techniques, careful adjustments to the deposition factors need to be made to reach the ideal stoichiometric ratio.^38,40^ This elemental composition analysis ensures that 2.5-Sb_2_S_3_ and 3.0-Sb_2_S_3_, Fig. 5(c) and (d), respectively, possess optimal composition (ideal stoichiometric ratio), along with superior morphological, structural, and *hk*1-oriented growth properties, as revealed by BF-TEM and SAED patterns. Fig. 5(e)–(h) show the elemental distribution of 1.5-Sb_2_S_3_, 2.0-Sb_2_S_3_, 2.5-Sb_2_S_3_ and 3.0-Sb_2_S_3_, respectively. It is worth noting that a layer of platinum was deposited onto the Sb_2_S_3_ film before this measurement. These distribution mapping images confirm the uniform spread of Sb_2_S_3_ throughout the substrate. However, images in Fig. 5(g) and (h) show some flaws regarding the distribution of Sb and S. To its justification, the area uncovered by Sb and S in the image of Fig. 5(g) is due to that location being covered by an adhesive tape, which prevented the deposition of the solution. Other areas in the sample possessed a uniform distribution. The image in Fig. 5(h) shows a rather unequal distribution of Sb and S along the surface. This could be because this image corresponds to an edge of the sample; however, there were other locations present with more uniform spread out (data not shown). Furthermore, 2.5-Sb_2_S_3_ (Fig. 5(g)) seemed to possess the least amount of carbon residue. 3.0-Sb_2_S_3_ (Fig. 5(h)) was observed to have some carbon residue left from the precursor solution but less than 1.5-Sb_2_S_3_ and 2.0-Sb_2_S_3_ (data not shown). Regarding this, further rigorous annealing may generate carbon-free films across all the samples; however, this may lead to the development of undesirable grain boundaries at the micron scale. As all the samples were deposited and annealed under the same conditions (spin-coated at 6000 rpm for 30 seconds and annealed at 300 °C), it can be concluded that adding 2.5 ml of CS_2_ can generate an almost carbon-free, uniformly distributed Sb_2_S_3_ film.

### 3.5. Variable angle spectroscopic ellipsometry

**Fig. 6:**
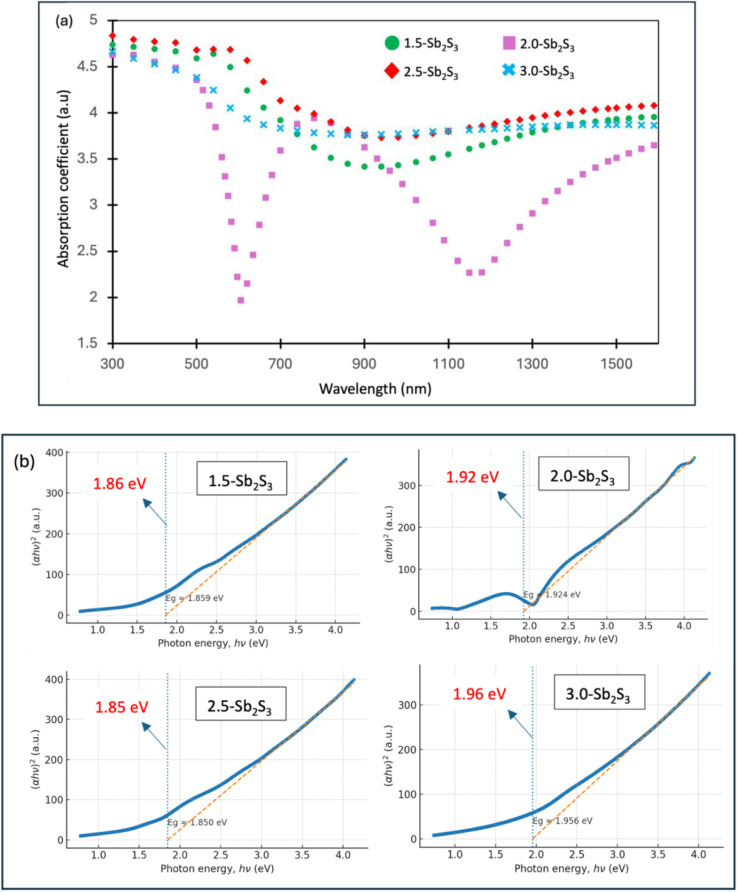
Variable angle spectroscopic ellipsometry (VASE) analysis of (a) absorption coefficient spectrum of 1.5-Sb_2_S_3_, 2.0-Sb_2_S_3_, 2.5-Sb_2_S_3_, and 3.0-Sb_2_S_3_. (b) Tauc plots of 1.5-Sb_2_S_3_, 2.0-Sb_2_S_3_, 2.5-Sb_2_S_3_, and 3.0-Sb_2_S_3_ showing the direct optical bandgap.

Variable Angle Spectroscopic Ellipsometry (VASE) was employed for the measurement of optical properties (absorption coefficient and bandgap) and constants (*n* and *k*) using the same fitting model. Fig. 6(a) shows the absorption coefficient spectrum across the wavelength range 300–1600 nm. The 2.5 ml CS_2_ used sample (2.5-Sb_2_S_3_), in red colour, displays the overall greatest absorption across the measured wavelengths. While the final stoichiometric ratio remains almost similar for all four samples, variation in CS_2_ significantly impacts the intermediate stages of film formation. The increased 2.5 ml CS_2_ concentration likely favours modulating precursor solubility, controlling nucleation kinetics and directing crystal growth planes. The superior light absorption for 2.5-Sb_2_S_3_ could therefore be attributed to its larger crystals and preferable *hk*1-oriented growth. The improved morphological uniformity of 2.5-Sb_2_S_3_ contributes further to reducing reflection losses, improving absorption. 2.0-Sb_2_S_3_ exhibits spectral fluctuations, which may be attributed to thickness inhomogeneities and surface roughness. Overall, these results show the potential optimization of absorption properties through strategic tuning of the precursor concentration.

The optical bandgaps of the films were evaluated using the equation given below (eqn 2):

2 (*αhv*)^*n*^ = *A*(*hv* − *E*_g_)

where *E*_g_ is the optical bandgap, *α* is the absorption coefficient, *v* is the frequency, *h* is the Planck constant, *A* is a material constant, and *n* = 2, 1/2, and 2/3, respectively, for allowed direct, allowed indirect, and forbidden direct transitions.

Allowed direct transition (*n =* 2) was used to obtain the bandgap values as they generated a smooth linear fit to the graph, and the corresponding Tauc plots are shown in Fig. 6(b). This confirms that all the fabricated Sb_2_S_3_ thin films are direct bandgap semiconductor materials, as has been reported before.^41^ From direct Tauc plot extrapolations, the optical bandgaps of the Sb_2_S_3_ thin films fall within a narrow range of 1.85–1.96 eV: 1.5-Sb_2_S_3_ – 1.86 eV, 2.0-Sb_2_S_3_ – 1.92 eV, 2.5-Sb_2_S_3_ – 1.85 eV, and 3.0-Sb_2_S_3_ – 1.96 eV. Rajpure and Bhosale^42^ reported a direct bandgap of 1.88 eV with a stoichiometric composition of 2 : 3 (Sb : S) which is very similar to our obtained values, also using a solution-processed approach. The range of values obtained in this research work also aligns well with other reports of Sb_2_S_3_ thin film.^20,36^ Slight changes in *E*_g_ values could be due to the combined effect of microstructural variations in crystal size, structural disorder, and thickness inhomogeneity. The minimal variations in *E*_g_ indicate that CS_2_ concentration has more influence on the film's crystal size and growth direction than on altering the intrinsic electronic structure. To further justify the narrow range of bandgap values, as all the samples were heated at the same temperature (300 °C), environment (vacuum) and duration (30 minutes), the films possessed similar bandgaps. Larger changes in bandgaps are often observed with changes in the annealing temperature or the end stoichiometric ratio, which in our case are almost constant across all the samples (1.5-Sb_2_S_3_, 2.0-Sb_2_S_3_, 2.5-Sb_2_S_3_, and 3.0-Sb_2_S_3_).^19,42^ Large changes in *E*_g_ can be obtained by varying electrodeposited potentials, with Al_2_Te_3_ having the *E*_g_ range of (2.26–2.68 eV), much higher than the reported values of Sb_2_S_3_ (1.85–1.96 eV) here.^43^

**Fig. 7:**
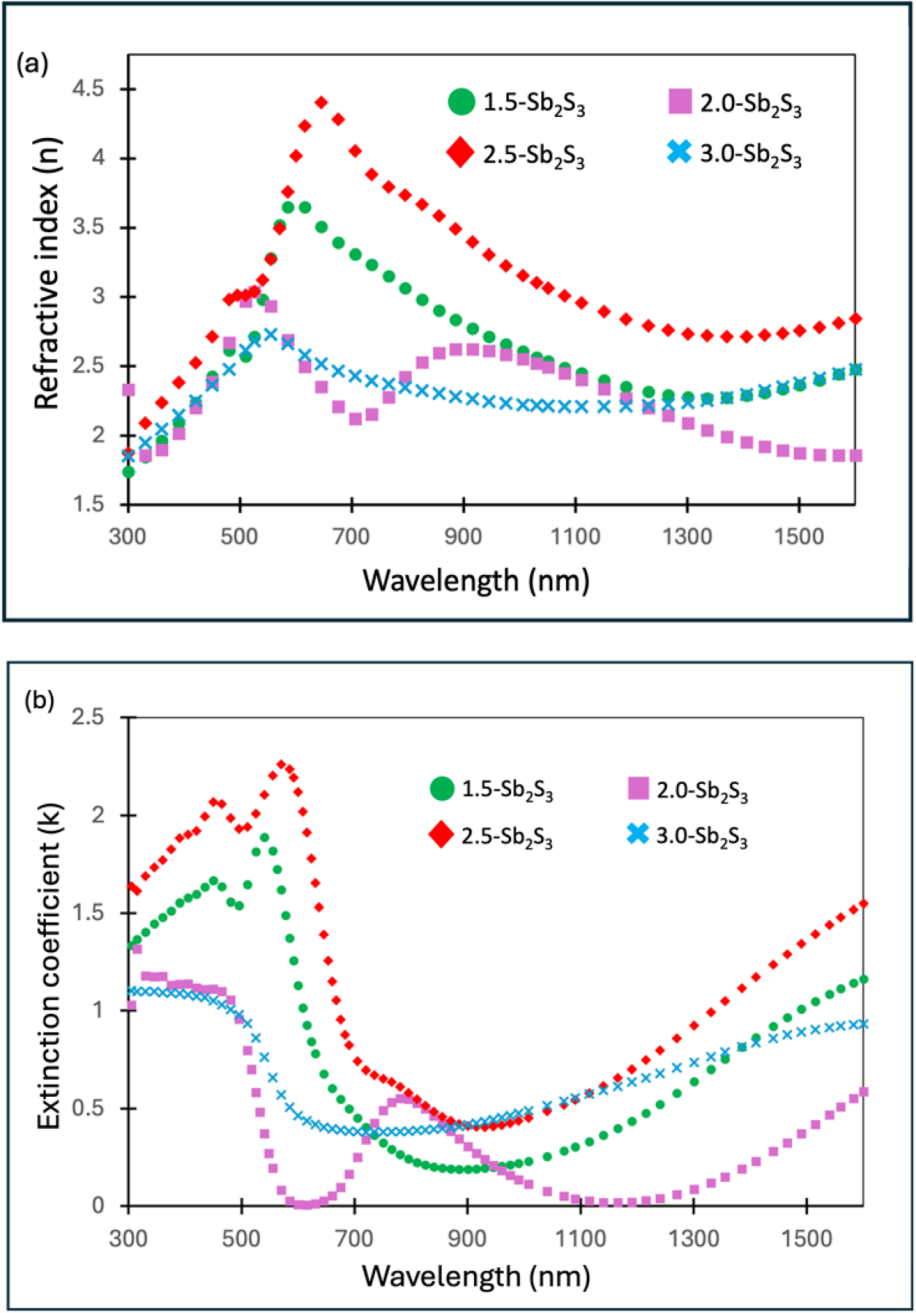
Variable angle spectroscopic ellipsometry (VASE) analysis of (a) the refractive index (*n*) spectrum of 1.5-Sb_2_S_3_, 2.0-Sb_2_S_3_, 2.5-Sb_2_S_3_, and 3.0-Sb_2_S_3_; (b) extinction coefficients (*k*) of 1.5-Sb_2_S_3_, 2.0-Sb_2_S_3_, 2.5-Sb_2_S_3_, and 3.0-Sb_2_S_3_.

In recent research advances, it has become increasingly important to optimize optical constants (refractive index and extinction coefficient) for the innovation of new opto-electronic devices.^44^ Enhancing the refractive index of Sb_2_S_3_ receives great importance due to its potential application in high-reflecting dielectric films.^19^Fig. 7(a) and (b) show the refractive index (*n*) and extinction coefficient (*k*) of the Sb_2_S_3_ film, respectively. 2.5-Sb_2_S_3_ (2.5 ml CS_2_) was observed to achieve the highest overall refractive index among the four samples over the wide wavelength range from 300 nm to 1600 nm. The obtained high *n* value of 2.5-Sb_2_S_3_ could be correlated to larger nanocrystals, as shown previously in bright-field TEM, combined with film compactness. The enhanced refractive index value obtained in this research work due to the strategic tuning of the precursor concentration is found to be higher than other reported values of Sb_2_S_3_.^16,19,45–47^ Moreover, some reported research studies show similar refractive index values as obtained here, but they utilized a more expensive non-solution based approach.^44,48,49^ Hence, it could be concluded that the proposed strategic tuning of the precursor concentration can result in a much higher refractive index (*n*) value than the usual. The extinction coefficient (*k*) quantifies how effectively a material absorbs light at a specific wavelength and is linked to the absorption coefficient (*α*) (eqn (3)).^50^ A greater absorption coefficient would generate a greater extinction coefficient (*k*) value.3
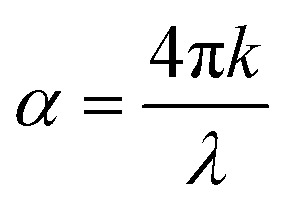
Our obtained experimental results of 2.5-Sb_2_S_3_ agree with this statement, achieving the overall highest *α* and *k* among all the samples. Fig. 7(b) shows the extinction coefficient (*k*) spectrum over the wavelength ranging from 300 to 1600 nm. As can be clearly observed from the plots of the curves, 2.5-Sb_2_S_3_ possesses the highest overall extinction coefficient (*k*) values. Such properties are excellent for applications that require efficient light harvesting in thin layers (∼100 nm), such as photovoltaics and photodetectors. The superior *k* value achieved by 2.5-Sb_2_S_3_ could correlate with a denser packed film with improved surface morphology and preferred orientation (*hk*1) of the crystal planes coupled together. The reported *k* value of 2.5-Sb_2_S_3_ in this research work is higher compared to other reported values of Sb_2_S_3_ films.^46,49^ Extinction coefficient (*k*) values reported by Medina-Montes *et al.*^48^ are similar to the *k* values that we have achieved for 2.5-Sb_2_S_3_ over the visible to near-infrared spectrum. Hence, this concludes that strategic tuning of the precursor concentration can effectively enhance the extinction coefficient (*k*) values.

## 4. Conclusion

Comprehensive structural, compositional, and optical investigations confirm the successful synthesis of Sb_2_S_3_ thin films with tuneable properties through controlled variation of the CS_2_ precursor concentration. Bright-field TEM images revealed that 2.5-Sb_2_S_3_ (precursor solution synthesized with 2.5 ml CS_2_) obtained the largest nanocrystals (∼120 nm). High-resolution TEM images along with FFT-diffractogram images revealed the pure crystalline phase with clear lattice fringes and without any evident defects. SEM images of all the samples confirmed the overall compact, homogeneous surface morphology with the absence of any dense grain boundary location across all the fabricated films, at the micron scale. SAED ring patterns revealed the presence of dense diffraction spot regions, which indicated the existence of smaller crystals. The Miller indices, calculated from the *d*-spacing of the SAED rings, helped identify the crystal planes. The texture coefficient (*hk*1/*hk*0) measurements revealed the superior *hk*1-oriented growth of 2.5-Sb_2_S_3_, obtaining the highest *hk*1/*hk*0 texture coefficient of 2.62. EDS measurements revealed the presence of the ideal stoichiometric ratio (Sb : S, 2 : 3) across all the samples, with 2.5-Sb_2_S_3_ being almost carbon-free. These structural, compositional observations directly correlated with the optical behaviour extracted from VASE measurements, where all samples exhibited direct band gaps in the range of 1.85–1.96 eV, in agreement with reported values for Sb_2_S_3_. Importantly, a clear distinction emerged among the samples: the 2.5 ml CS_2_ composition consistently showed superior optical performance, with the highest absorption coefficient, enhanced refractive index (*n*), and extinction coefficient (*k*), indicating efficient light–matter interaction. In contrast, films prepared with either lower or higher CS_2_ concentrations exhibited weaker optical responses, suggesting that precursor chemistry plays a decisive role in balancing nucleation and growth kinetics. Taken together, these results identify the 2.5 ml CS_2_-derived Sb_2_S_3_ thin film as the optimum recipe, combining enlarged crystal size and favourable *hk*1-oriented growth, to obtain superior optical response and highlight its strong potential for integration into next-generation photovoltaic and optoelectronic devices.

## Author contributions

Md Abrar Faisal Hossain: conceptualization, formal analysis, investigation, methodology, visualization, writing – original draft, writing – review & editing. Kyota Shirai: investigation, methodology, project administration, resources. Masayuki Shimojo: funding acquisition, supervision, writing – review & editing.

## Conflicts of interest

The authors confirm that there are no conflict of interests.

## Data Availability

The authors confirm that the data supporting the findings of this research work can be found in this article. ImageJ software was used for analysing TEM images and indexing SAED patterns. Microsoft Excel was used for plotting the graphs. Microsoft Word was used for writing the research article.
